# Methadone maintenance treatment alters couplings of default mode and salience networks in individuals with heroin use disorder: A longitudinal self-controlled resting-state fMRI study

**DOI:** 10.3389/fpsyt.2023.1132407

**Published:** 2023-04-17

**Authors:** Jiajie Chen, Yongbin Li, Shu Wang, Wei Li, Yan Liu, Long Jin, Zhe Li, Jia Zhu, Fan Wang, Wei Liu, Jiuhua Xue, Hong Shi, Wei Wang, Chenwang Jin, Qiang Li

**Affiliations:** ^1^Department of Radiology, The First Affiliated Hospital of Xi’an Jiaotong University, Xi’an, Shaanxi, China; ^2^Department of Radiology, Tangdu Hospital, Fourth Military Medical University, Xi'an, Shaanxi, China; ^3^Department of Radiology, Xi'an No. 1 Hospital, Xi'an, Shaanxi, China; ^4^Biomedical Engineering, School of Life Science and Technology, Xi'an Jiao Tong University, Xi'an, Shaanxi, China

**Keywords:** methadone maintenance treatment, large-scale networks, withdrawal symptoms, heroin, addiction, functional magnetic resonance imaging

## Abstract

**Background:**

Methadone maintenance treatment (MMT) is a common treatment for heroin use disorder (HUD). Although individuals with HUD have been reported to show impaired coupling among the salience network (SN), executive control network (ECN), and default mode network (DMN), the effects of MMT on the coupling among three large-scale networks in individuals with HUD remains unclear.

**Methods:**

Thirty-seven individuals with HUD undergoing MMT and 57 healthy controls were recruited. The longitudinal one-year follow-up study aimed to evaluate the effects of methadone on anxiety, depression, withdrawal symptoms and craving and number of relapse, and brain function (SN, DMN and bilateral ECN) in relation to heroin dependence. The changes in psychological characteristics and the coupling among large-scale networks after 1 year of MMT were analyzed. The associations between the changes in coupling among large-scale networks and psychological characteristics and the methadone dose were also examined.

**Results:**

After 1 year of MMT, individuals with HUD showed a reduction in the withdrawal symptom score. The number of relapses was negatively correlated with the methadone dose over 1 year. The functional connectivity between the medial prefrontal cortex (mPFC) and the left middle temporal gyrus (MTG; both key nodes of the DMN) was increased, and the connectivities between the mPFC and the anterior insular and middle frontal gyrus (key nodes of the SN) were also increased. The mPFC-left MTG connectivity was negatively correlated with the withdrawal symptom score.

**Conclusion:**

Long-term MMT enhanced the connectivity within the DMN which might be related to reduced withdrawal symptoms, and that between the DMN and SN which might be related to increase in salience values of heroin cues in individuals with HUD. Long-term MMT may be a double-edged sword in treatment for HUD.

## Introduction

Heroin use disorder (HUD) is characterized by excessive heroin-seeking and heroin-consumption behaviors despite patients’ knowledge that these behaviors are harmful for their physical and mental health (1). Individuals with HUD often show a chronically relapsing cycle of withdrawal and craving despite the substantial adverse consequences. Approximately 24 to 43% of individuals with HUD had anxiety symptoms (2), and heroin also may contribute to major depressive disorder in HUDs (3). The methadone maintenance treatment (MMT) program is one of the most effective treatments for heroin dependence. Behavioral studies have demonstrated that MMT effectively alleviates heroin dependence, reduces overdose fatality, improves the abstinence rate, and yields a better quality of life in individuals with HUD (4). Nevertheless, up to 53% of individuals with HUD do not maintain stable abstinence (5). Some studies have reported a gradual increase in the drop-out rate and relapse rate during MMT. The drop-out rate was 14% during the first year, 17% during the second year, and 22% during the third year (6). Relapse rates were even higher with 60% after 1 year (7). Moreover, MMT increases anxiety symptom among HUDs (8), which may lead to high suicide rate (9) and increased relapse rate (8). For individuals with HUD, the effect of MMT on the functional connectivity of brain is largely unknown, and neuroimaging methods may help characterize the mechanisms underlying the effects of MMT on the brain.

Numerous studies have shown different effects of MMT. Long-term MMT could increase the local activity in right dorsolateral frontal cortex and bilateral parietal cortex and might help restore executive control function toward that of healthy controls for individuals with HUD (10). However, some studies have implied that long-term MMT program might lead to impaired structure and function of parts of brain for individuals with HUD. A longitudinal study found that individuals with HUD show a smaller gray matter volume in insula, cingulate cortex, caudate nucleus after 1 year of MMT (11). Heroin relapsers undergoing MMT program showed lower white matter integrity in the right retrolenticular part, left anterior and posterior limb of internal capsule, bilateral anterior corona radiata relative to abstainers (12). Compared with healthy controls, the individuals with HUD undergoing MMT program had a lower interhemispheric insular functional connectivity (13). Furthermore, a higher methadone dose was associated with a smaller globus pallidus in the MMT group (14). However, these studies were cross-sectional, and the diversity of results could have been attributed to the presence of many variable and complex factors related to the research participants. Thus, a longitudinal self-controlled study may yield objective conclusions. However, few self-controlled longitudinal functional neuroimaging studies have focused on the effect of MMT on the functional connectivity characteristics of the brain in individuals with HUD.

The increased interest in understanding the mechanisms underlying intrinsic fluctuations in the ongoing resting activity among large-scale brain functional networks is relevant to cognitive dysfunctions during MMT in individuals with HUD. In this regard, a triple-network model composed of the salience network (SN), executive control network (ECN), and default mode network (DMN) has received the most attention. The current view suggests that the ECN and DMN show anticorrelation at rest, and the SN performs the critical role of adjusting brain activity between the DMN and ECN, driving attention resources into internal or external stimuli (15). One study on HUD suggested that the number of relapses are positive associated with enhanced SN-DMN coupling and negative associated with decreased left ECN-DMN coupling (16). Our previous findings preliminarily suggest that MMT could increase the coupling of the SN and the bilateral ECN in individuals with HUD compared to healthy controls and untreated HUDs (17). However, this study is cross-sectional and lacks longitudinal in-depth exploration. Up to now, little is known about the mechanisms underlying the influence of long-term MMT on coupling among the SN, DMN, and ECN in individuals with HUD.

In the current study, we recruited a cohort of individuals with HUD who were undergoing stable MMT as a part of a longitudinal self-controlled resting-state fMRI study. We also recruited a cohort of healthy individuals as controls. We hypothesized that (1) after 1 year of MMT, individuals with HUD would show lower functional connectivity between the SN and DMN and stronger functional connectivity between the SN and ECN compared with baseline; (2) longer-term MMT is helpful in decreasing the craving for heroin and relieving negative symptoms such as withdrawal symptoms; (3) the changes in connectivity would be associated with changes in negative symptoms and craving for heroin.

## Materials and methods

### Study design

This observational, prospective, longitudinal one-year follow-up study aimed to evaluate the effects of methadone on anxiety, depression, withdrawal symptoms and craving and relapse behavior, and brain function in relation to HUD. Individuals with HUD taking stable doses of methadone were recruited for this study, and their psychological characteristics and resting-state fMRI data were collected at baseline and 1 year later, respectively. During this one-year follow-up period, all individuals with HUD underwent monthly random urine testing. Urine tests were considered positive if the concentration of drugs in the urine exceeded the cut-off threshold of 300 ng/ml for morphine, 1,000 ng/ml for methamphetamine and 1,000 ng/ml for ketamine. This was done to detect the presence of relapse behavior in individuals with HUDs during MMT program. Individuals in the healthy control group were measured only once and were not followed up. The study was approved by the Ethics Committee of Tangdu hospital. All participants provided informed consent prior to the study.

### Participants

Patients undergoing MMT who met the following criteria were enrolled from a heroin treatment program in Baqiao District, Xi ‘an: (1) diagnosed as showing HUD (for at least 6 months) based on the Diagnostic and Statistical Manual of Mental Disorders (Fifth Edition), (2) receiving a stable dosage of methadone for at least 3 months, and (3) right-handed. Healthy volunteers from the nearby community around Tangdu Hospital were recruited as the healthy control (HC) group. All participants who showed any of the following findings were excluded: (1) a history of substance use disorder except heroin and nicotine, (2) a diagnosis of past or current brain disease, especially brain injury and neurological disorders, (3) inability to complete psychological and behavioral measurements and MRI examinations, and (4) contraindications for MRI scanning (such as claustrophobia, hyperpyrexia, and metal implants). Seventy-four patients undergoing MMT and 60 HCs were initially enrolled in the study. Part of the data came from our previous study (17).

### Measures

Demographic characteristics included sex, age, years of education, history of smoking, and history of heroin and methadone usage. Psychological scores are assessed by self-report. Psychological assessments were taken twice for individuals with HUDs, at the time of enrollment and 1 year after MMT program. Psychological assessments for healthy controls were taken only once at enrollment and were not followed up. Psychological characteristics included anxiety (measured with Hamilton Anxiety Scale-14, HAMA-14) (18), depression (evaluated with Beck Depression Inventory II, BDI-II) (19), and withdrawal symptoms (assessed with a published scale for heroin dependent individuals) (20) as well as pre-cue and post-cue craving scores. The pre-cue craving assessment was completed prior to MRI data acquisition. After resting-state functional MRI data acquisition, the drug cue-induced craving task was administered, and the post-cue craving score was evaluated again. Individuals with HUD underwent urine testing for morphine, methamphetamine and ketamine once a month during the one-year follow-up study.

The craving scores for heroin was assessed using an event-related picture cue craving paradigm (20–22). This task included 24 heroin-related (drug scenes, drug paraphernalia, etc.) and 24 neutral control (bus, tree, etc.) cue pictures. Each picture was showed for 2 s with an interval of 4–12 s in pseudorandom mode through a computer. Each subject self-reported a score (0–10) for heroin craving before and after the task on a visual analog scale (VAS), within which “0” for no craving and “10” for very severe craving.

### Magnetic resonance imaging

All participants were required to have a negative urine test for morphine, ketamine, and methamphetamine on the day of the MRI scan, and individuals with HUD took methadone after the MRI scan. A General Electric Signa Excite HD 3.0 T MRI scanner equipped with a standard eight-channel head coil at Tangdu Hospital was used for functional magnetic resonance imaging (fMRI). All participants were instructed to relax and quietly view a white cross in the background through a mirror mounted on the head coil during the resting-state fMRI scan. To help participants acclimatize to the MRI scanning environment, mock scanning was conducted for 1 min without data collection. Subsequently, resting-state images were collected during the formal scan for 5 min.

Resting-state fMRI was performed by a gradient-echo echo-planar imaging sequence with the following parameters: repetition time = 2000 ms, echo time = 30 ms, flip angle = 90°, thickness = 4 mm, spacing = 0 mm, number of slices = 32, field of view = 256 mm × 256 mm, matrix = 64 × 64, number of excitation = 1, and 150 volumes. 3D high-resolution anatomical images (T1-weighted sequence) were acquired for the resting-state imaging registration by a fast spoiled phase gradient-echo sequence. Its parameters are as follow: repetition time = 7.8 ms, echo time = 3.0 ms, flip angle = 20°, inversion time = 450 ms, thickness = 1 mm, number of slices = 166, spacing = 0 mm, FOV = 256 mm × 256 mm, matrix = 256 × 256, number of excitation = 1. The detailed scanning parameters were the same as in a previous study (17). All images were evaluated by two neuroradiologists to eliminate cases showing structural abnormalities. However, no participant was excluded on the basis of this criterion.

### Image data processing

#### Preprocessing

The preprocessing was performed using DPABI,^1^ SPM12,^2^ and MATLAB 8.1^3^ for neuroimaging. The resting-state fMRI dates for each participant were corrected for slice timing to eliminate time differences caused by multi-slice interval imaging. For each participant, images were then spatially realigned to the first scan of the series. Motion artifacts of all participants were monitored through a realignment parameter. Subjects with the maximum head motion exceeding 3 mm in translation or 3.0° in rotation were removed. To accurately locate the functional brain area, the functional image was co-registered to the respective high-resolution anatomical image (T1 weighted sequence). The brain tissue is divided into gray matter, white matter, and cerebrospinal fluid with a new segmentation method in structural image. Subsequently, the nuisance covariates, namely, 24 head motion parameters and white matter and cerebrospinal fluid signals, were regressed out. The original space of functional brain was registered to the standard anatomical space (Montreal Neurological Institute, MNI) and resample to 3 × 3 × 3 mm, addressing differences in brain morphology among subjects. Dates are spatially smoothed using a Gaussian filter with a 6-mm full-width at half maximum kernel to improve the signal-to-noise ratio (SNR) after normalization. At the end of preprocessing, linear detrend and band-pass filtering (0.01–0.1 Hz) was applied to the images.

#### Identification of the DMN, ECN, and SN

Independent component analysis (ICA) was performed on the smoothed data of all participants by using the GIFT 4.0 toolbox.^4^ Most parameters and procedures were set to the default values. First, the GIFT software was used to estimate the number of independent components and the number of ICA components were set as 25. Second, Principal Component Analysis (PCA) was used to reduce the dimensionality in two steps (individual level and group level). At the individual level, the dimensionality of a single subject was used to reduce by PCA, and 38 principal components were retained. At the group level, 25 principal components of all subjects were dimensionally reduced. Then, based on the independent components obtained at the group level, a back reconstruction of the data to individual subject-independent components was also conducted to examine the reproducibility of the components detected. All components were identified through the back reconstruction. ICA was repeated 40 times by running randinit and bootstrap mode in the ICASSO toolbox to ensure repeatable and stable results. Referring to the resting state network reported in previous studies, the components were defined based on structural and functional properties. The spatial z-map of reconstructed components was used to calculate range of brain regions within each independent network. The SN, bilateral ECN, and DMN were identified by one-sample *t*-test [Gaussian Random Field (GRF) correction, voxel-*p* < 0.001, cluster-*p* < 0.05].

**Figure 1:**
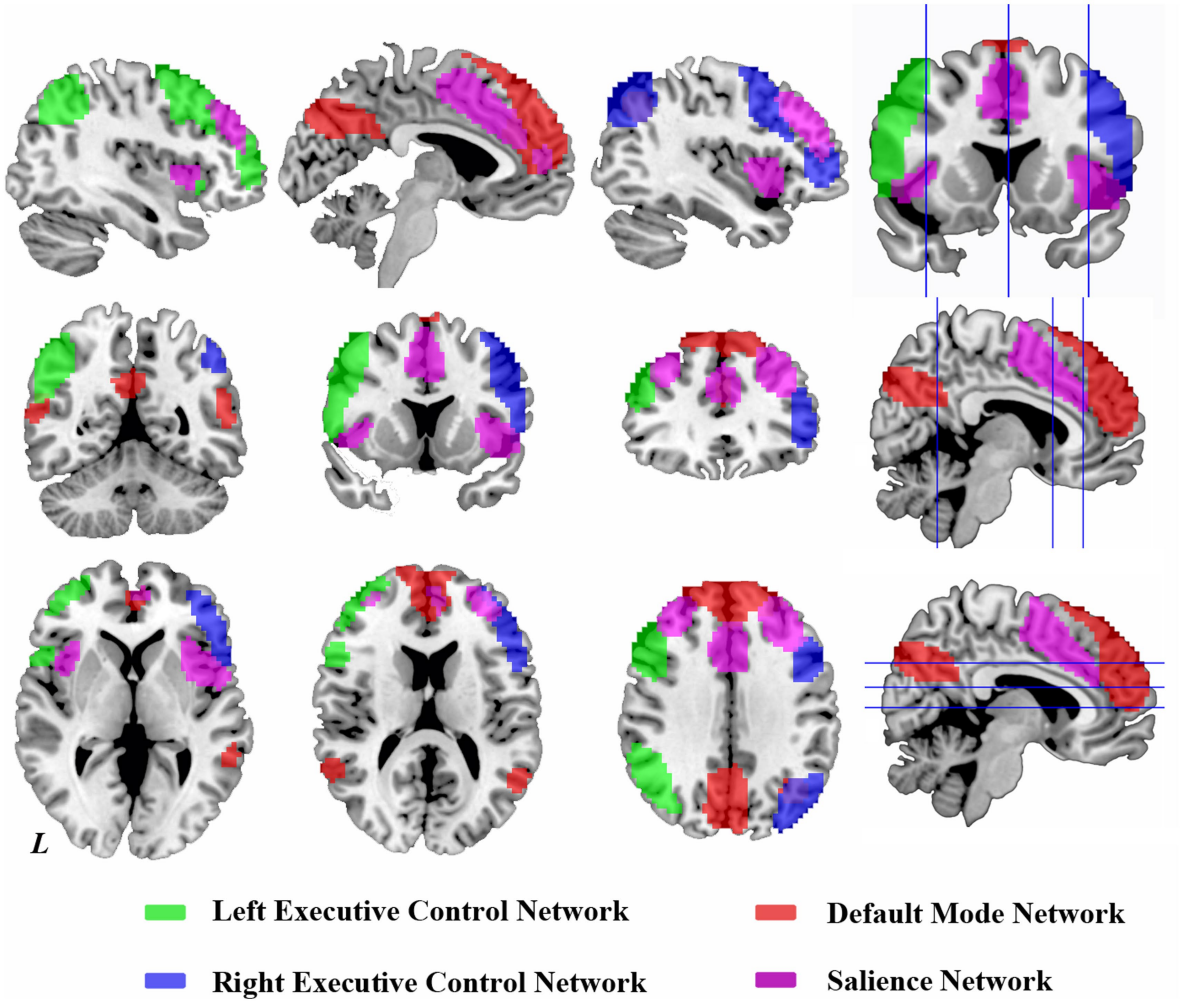
Brain networks revealed by the group-level independent component analysis.

The SN included the dorsal anterior cingulate cortex (dACC), bilateral anterior insula (AI), and middle frontal gyrus (MFG) (23). The DMN included the medial prefrontal cortex (mPFC), precuneus/posterior cingulate cortex (PCC), and bilateral middle temporal gyrus (MTG). The left ECN consisted of the left dorsolateral prefrontal cortex (dlPFC) and posterior parietal cortex (PPC). The right ECN included the right dlPFC and PPC (Table 1 and Figure 1). We performed principal components analysis on all individuals, including HC and MMT individuals, and determined DMN, SN, and ECN by within-group one-sample t-test. The identified map served as a mask for functional connectivity of the brain networks.

**Table 1:** Large-scale networks from the group independent component analysis.

Network	Brain area	Side of the brain (R/L)	*X*	*Y*	*Z*	Peak intensity	Cluster size (mm^3^)	Cluster size (voxels)
DMN	MPFC		0	55	19	28.79	50,355	1865
	PCC		0	−70	30	33.06	45,063	1,669
	MTG	R	57	−51	15	7.55	1971	73
	MTG	L	−39	−63	42	11.13	11,421	423
LECN	dlPFC	L	−51	21	21	28.63	48,033	1779
	PPC	L	−54	−54	27	14.33	14,904	552
RECN	dlPFC	R	48	18	39	16.14	35,316	1,308
	PPC	R	48	−54	42	25.69	20,088	744
SN	ACC		0	35	29	26.09	24,273	899
	MFG	L	−30	39	36	26.71	19,143	709
	AI	L	−39	12	0	16.46	9,504	352
	MFG	R	33	42	33	22.47	9,450	350
	AI	R	39	15	0	10.5	3,213	119

SN, salience network; DMN, default mode network; ECN, executive control network; dACC, dorsal anterior cingulate cortex; mPFC, medial prefrontal cortex; PCC, posterior cingulate cortex; dlPFC, dorsolateral prefrontal cortex; AI, anterior insula; MTG, middle temporal gyrus; MFG, middle frontal gyrus.

#### Definition of regions of interest and functional connectivity

To analyze the group differences in connectivity among the SN, bilateral ECN, and DMN, we selected the peak voxel of each key region in the identified large brain network as the centers of spherical regions of interest (ROIs; radius = 6 mm). The voxel-wise functional connectivity analyses were performed on band pass-filtered data based on each ROI within all the defined SN, bilateral ECN, and DMN. Pearson correlation coefficients were used to estimate the functional connectivity among the SN, bilateral ECN, and DMN [16; 17] with the DPABI software. The paired *t*-test was performed to test differences in functional connectivity based on each ROI of the SN, bilateral ECN, and DMN between the Baseline and one-year follow-up groups with the DPABI software (GRF correction, voxel-*p* < 0.001, and cluster-*p* < 0.05).

### Correlation analysis

Pearson correlation analyses were performed between the functional connectivity strength of differential regions and psychological characteristics (such as depression, anxiety, withdrawal symptoms, pre-cue-induced craving scores and post-cue-induced craving scores) at the baseline and 1 year later. The correlation between the change in functional connectivity strength of differential regions and the total methadone dose during the year was analyzed. The correlation between the psychological characteristics and number of relapses were also analyzed 1 year later. The correlation results were corrected for multiple comparisons with the Bonferroni correction method.

## Results

### Demographics

For this longitudinal study, 74 patients receiving MMT were initially enrolled, of which 33 patients were lost to follow-up after 1 year. The resting-state fMRI data of four participants were excluded because of head movements. Thus, 37 patients receiving MMT were eventually enrolled in the study. Among the 60 HCs that were initially recruited, three were ruled out due to head movements or incomplete brain scans. Finally, 57 healthy controls were included in this study.

Table 2 summarizes the demographic and clinical characteristics of the MMT and HC groups. The MMT patients and HCs were well-matched for sex, age, education, history of smoking, anxiety symptom, and depression symptom (*p* > 0.05). The groups did not differ in mean head motion during the resting fMRI scan (*p* > 0.05).

**Table 2:** Demographic, heroin usage, and methadone usage characteristics of the Methadone maintenance treatment (MMT) and healthy control (HC) groups (mean ± standard deviation).

Participant characteristics	MMT (*N* = 37)	HC (*N* = 57)	*t*/*χ*^2^	*P*
Sex (male/female)^a^	33/4	52/5	−0.11	0.74
Age (years)	37.57 ± 7.50	35.63 ± 6.91	1.28	0.2
Years of education	9.43 ± 2.46	10.37 ± 2.35	−1.85	0.07
Years of smoking cigarettes	18.92 ± 8.11	16.14 ± 8.01	1.64	0.11
Cigarettes per day	18.89 ± 8.89	16.46 ± 9.47	1.25	0.22
Anxiety (HAMA)	9.27 ± 10.77	6.59 ± 3.29	1.47	0.15
Depression (BDI)	9.97 ± 8.78	7.25 ± 5.34	1.70	0.10
Mean FD_Power	0.16 ± 0.08	0.15 ± 0.10	0.62	0.54
Mean FD_Jenkinson	0.1 ± 0.04	0.09 ± 0.06	1.67	0.1
Duration of heroin usage (months)	113.94 ± 85.78	-	-	-
Amount of heroin used per day (g)	0.38 ± 0.4	-	-	-
Duration of methadone usage (months)	39.54 ± 19.38	-	-	-
Amount of methadone used per day (ml)	41.38 ± 19.87	-	-	-

HAMA, Hamilton Anxiety Scale; BDI, Beck Depression Inventory; FD, frame wise displacement. a: *x*^2^ The degree of freedom of the two-sample *t*-test is 92. The degree of freedom of the chi-square test is one.

### Psychological characteristics

**Figure 2:**
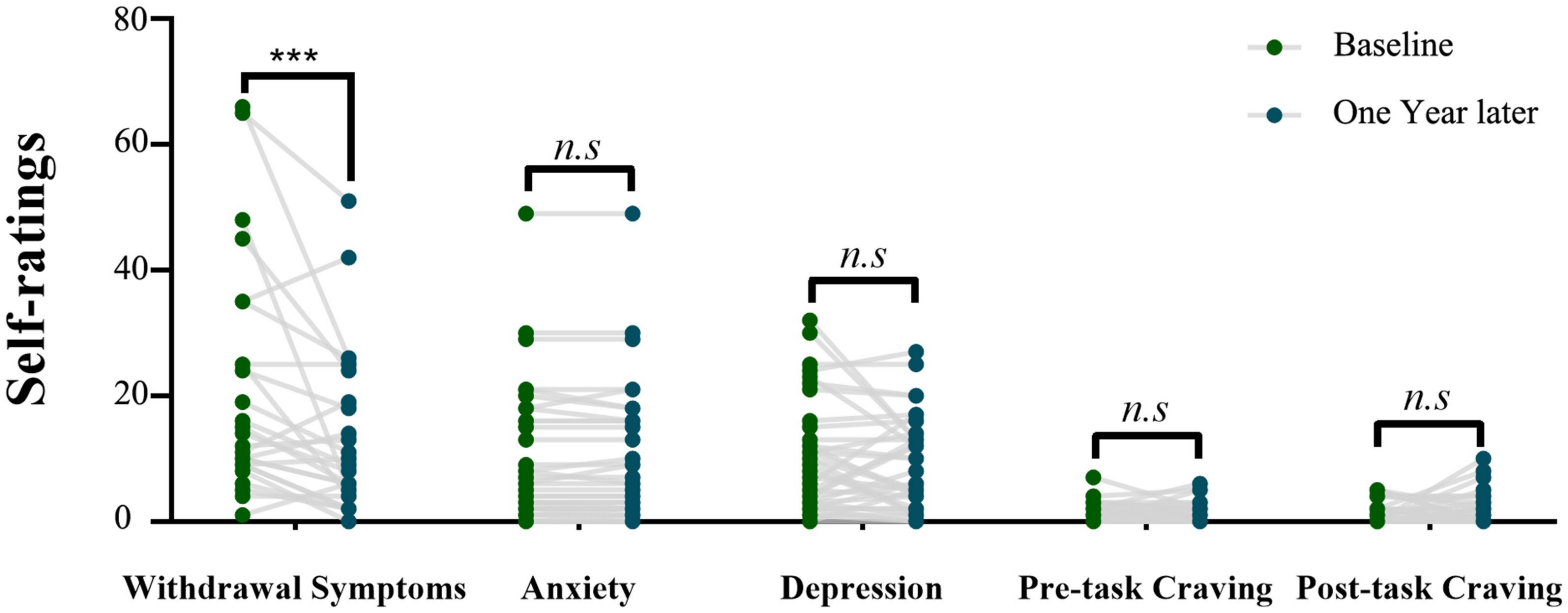
The scores of psychological variables of the individuals with HUD at the Baseline and 1 year later. Comparison between the two time points by using the paired-sample *t*-test (***p = 0.005).

In the self-assessment of opioid withdrawal symptom score, 31 individuals with HUD completed the evaluation for the first time. One year later, only 27 individuals with HUD completed the evaluation for the second time. During the one-year follow-up period, the opioid withdrawal symptom scores (mean ± standard error) of 27 individuals with HUD at the Baseline and 1 year later were 20.62 ± 3.54 and 13.12 ± 2.50, respectively. The individuals with HUD showed a significantly lower withdrawal symptom level (*n* = 27, *t*_(26)_ = 3.11, *p* = 0.005 Cohen’s dz. = 0.60) 1 year later (Figure 2).

The pre-cue-induced craving scores (mean ± standard error) of the baseline and 1 year later were 0.97 ± 0.25 and 1.19 ± 0.26, respectively, while the post-cue-induced craving scores (mean ± standard error) were 1.03 ± 0.28 and 1.73 ± 0.40, respectively. The pre- and post-cue-induced craving scores showed no significant changes during one-year follow-up (*t*_(36)_ = −0.85, *p* = 0.4; *t*_(36)_ *=* −1.5, *p* = 0.14; Figure 2). The anxiety symptom scores (mean ± standard error) at baseline and 1 year later were 9.27 ± 1.77 and 10.24 ± 1.54, respectively. The depression symptom scores (mean ± standard error) of Baseline and 1 year later were 9.97 ± 1.44 and 8.78 ± 1.21, respectively. The individuals with HUD showed no significant changes in the anxiety and depression symptom scores after one-year follow-up (*t*_(36)_ = 1.14, *p* = 0.26; *t*_(36)_ = −0.94, *p* = 0.35; Figure 2).

### Relapse

**Figure 3:**
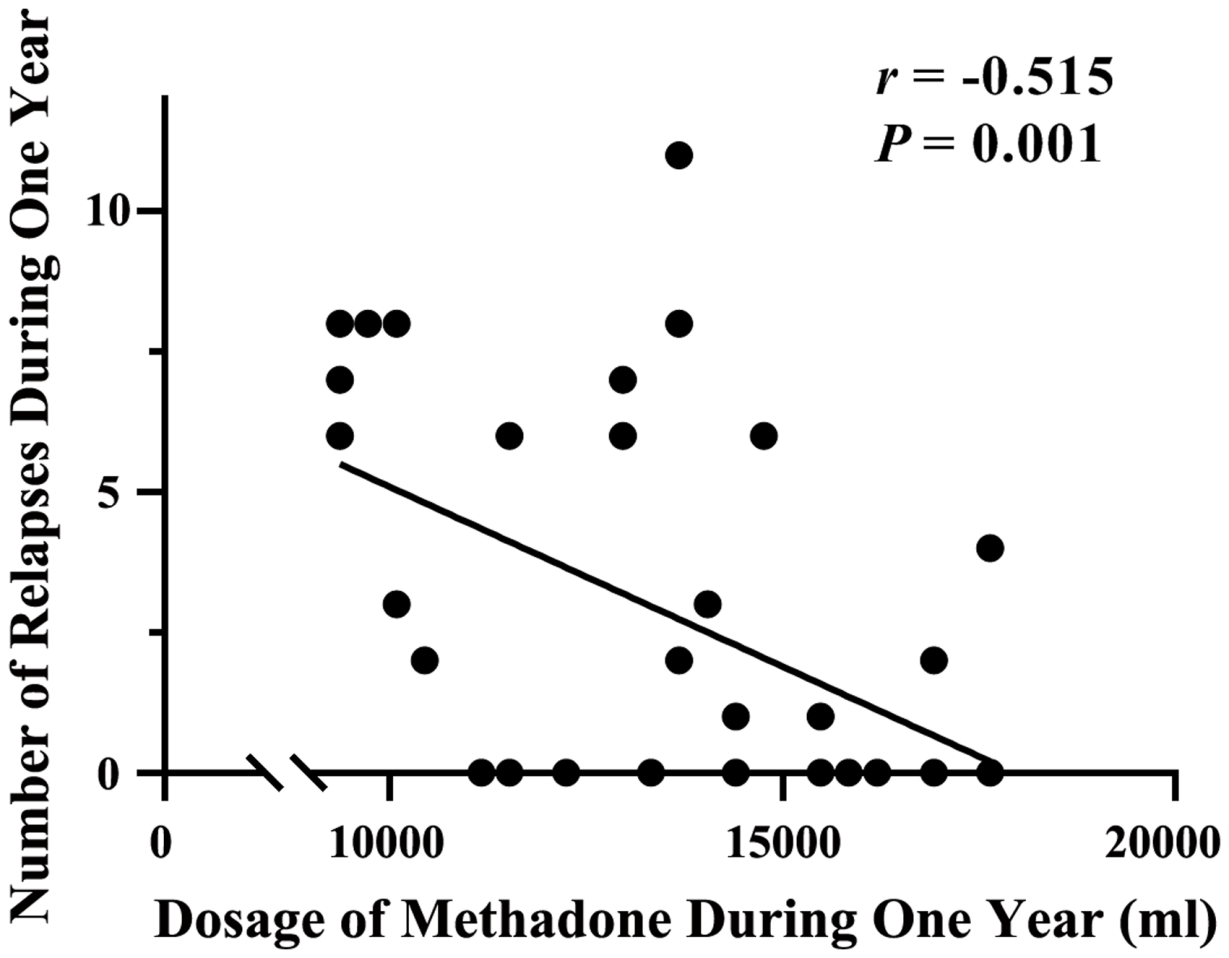
Correlation between the number of relapses and total methadone dose during the one-year follow-up period.

During the one-year follow-up period, 19 of the 37 patients undergoing MMT (51.35%) relapsed. The number of occurrences of relapse within a year was 5.25 ± 2.83 (range, 1–11). The number of relapses was negatively related to the total methadone dose in 1 year (*r* = −0.515, *p* = 0.001, Bonferroni corrected *p* = 0.05/5 = 0.01; Figure 3).

### Brain network connectivity

**Figure 4:**
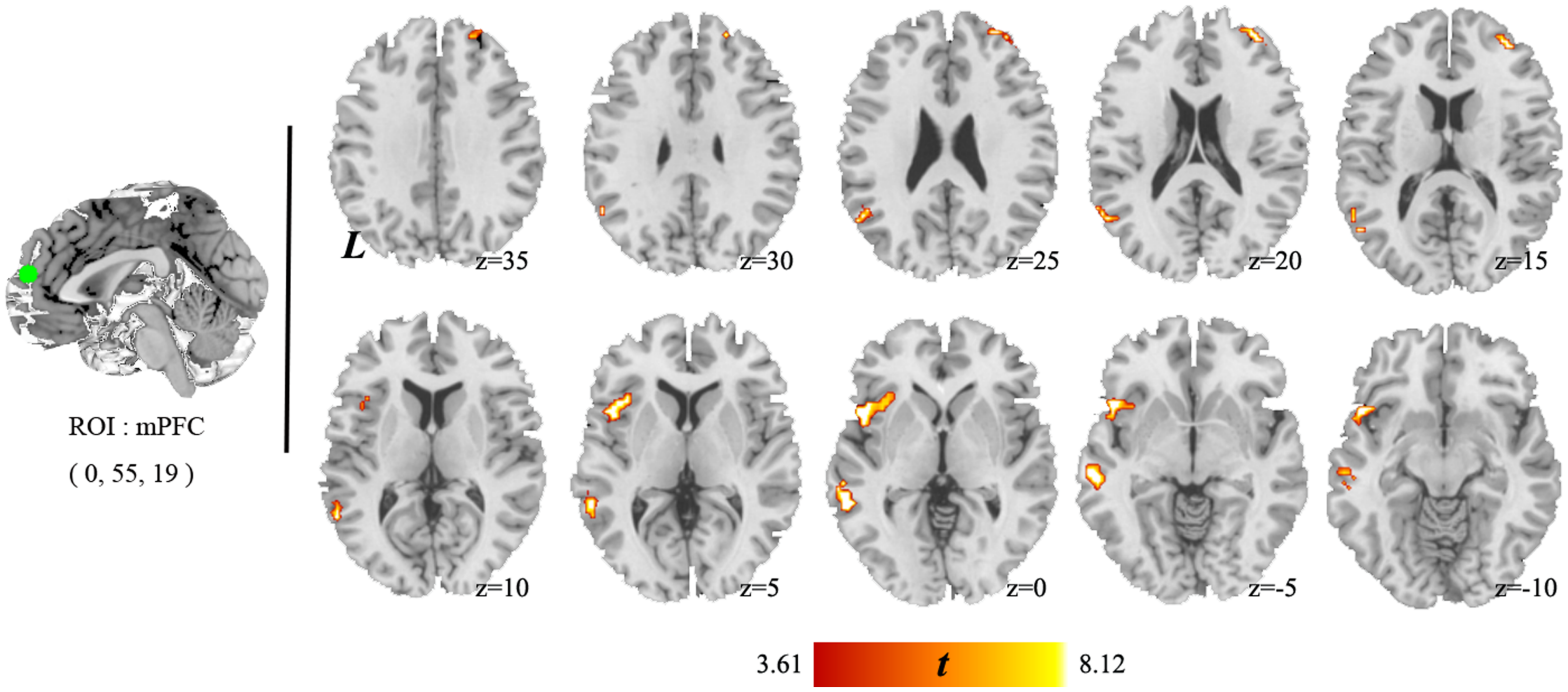
Brain areas showing significant changes in mPFC connectivity between baseline and 1 year later. The individuals with HUD showed higher functional connectivity in the left middle temporal gyrus, left anterior insula, and right middle temporal gyrus after 1 year follow-up.

The paired *t*-test results showed that only the change in connectivity based on the mPFC (a key node of DMN), which included the left MTG and PPC and the right MFG among the individuals with HUD between baseline and 1 year later (Figure 4), survived the GRF correction (*voxel-p* < 0.001, *cluster-p* < 0.05). This is a longitudinal self-controlled study to observe the effects of methadone on brain connectivity of individuals with HUD. Since it is a self-controlled, longitudinal, observational study, the HC group was only used as a reference control. Therefore, we did not conduct cross-sectional comparison in connectivity between HC and Baseline and one-year follow-up MMT groups.

#### Within-DMN connectivity

**Figure 5:**
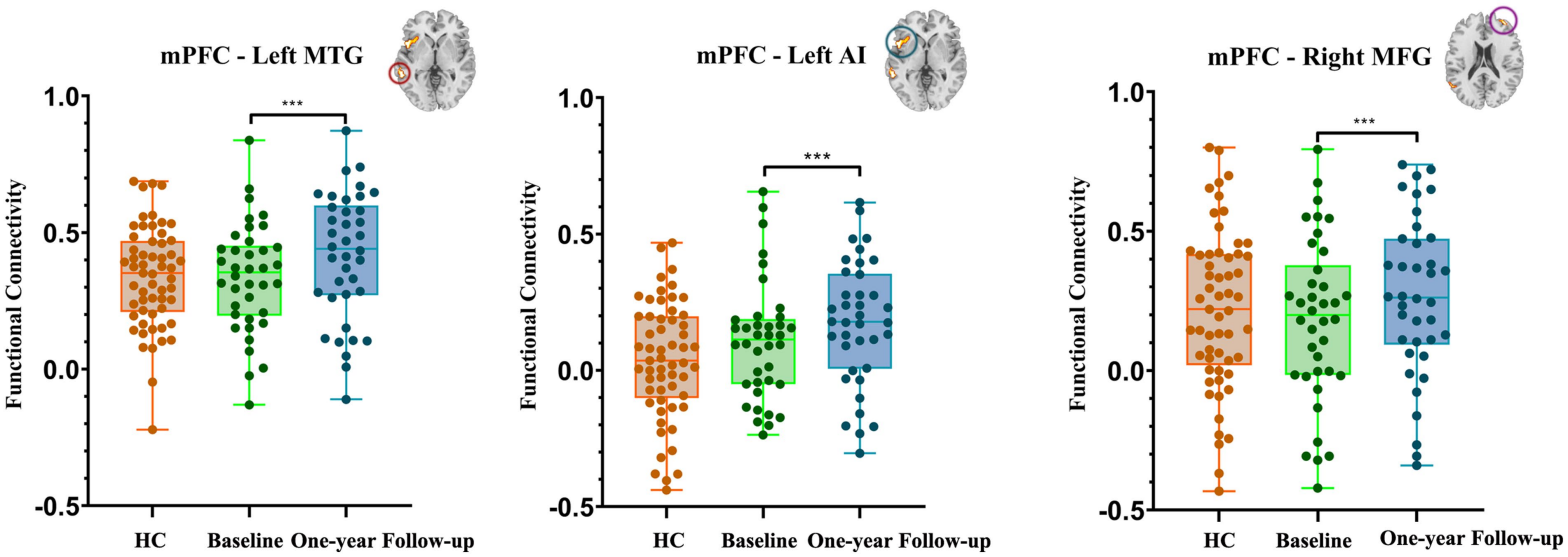
Bar chart of the post-hoc results related to the changes in functional connectivity based on mPFC after one-year follow-up. The data of the HC group is presented as control data (HC, healthy controls; mPFC, medial prefrontal cortex; MTG, middle temporal gyrus; AI, anterior insula; MFG, middle temporal gyrus. ***p < 0.001).

In comparison with the baseline, the individuals with HUD under MMT showed significantly higher functional connectivity between the mPFC and the left MTG (key nodes of the DMN) 1 year later (*t*_(36)_ = 7.18, *voxel-p* < 0.001, *cluster-p* < 0.05, corrected with GRF, Cohen’s dz. = 1.18; Table 3, Figure 5).

**Table 3:** Changes in functional connectivity based on medial prefrontal cortex (mPFC) in the salience network (SN), executive control network (ECN), and default mode network (DMN) in individuals with heroin use disorder (HUD) after 1 year follow-up.

Brain area	Peak Coordinate			Peak Intensity (*t*-value)	Cluster size (voxels)	Cluster size (mm^3^)
	*X*	*Y*	*Z*			
Right MTG	−51	−57	24	7.13	112	3,024
Left AI	−48	6	3	7.18	105	2,835
Left MFG	33	51	21	6.56	32	864

#### DMN-SN connectivity

In comparison with the Baseline, the individuals with HUD under MMT showed significantly higher functional connectivity between the mPFC and the left AI and right MFG (nodes of the SN) 1 year later (*t*_(36)_ = 7.13*, t*_(36)_ = 7.02*, voxel-p* < 0.001, *cluster-p* < 0.05, corrected with GRF, Cohen’s dz. = 1.17; Table 3, Figure 5).

### Correlation between brain connectivity and psychology

**Figure 6:**
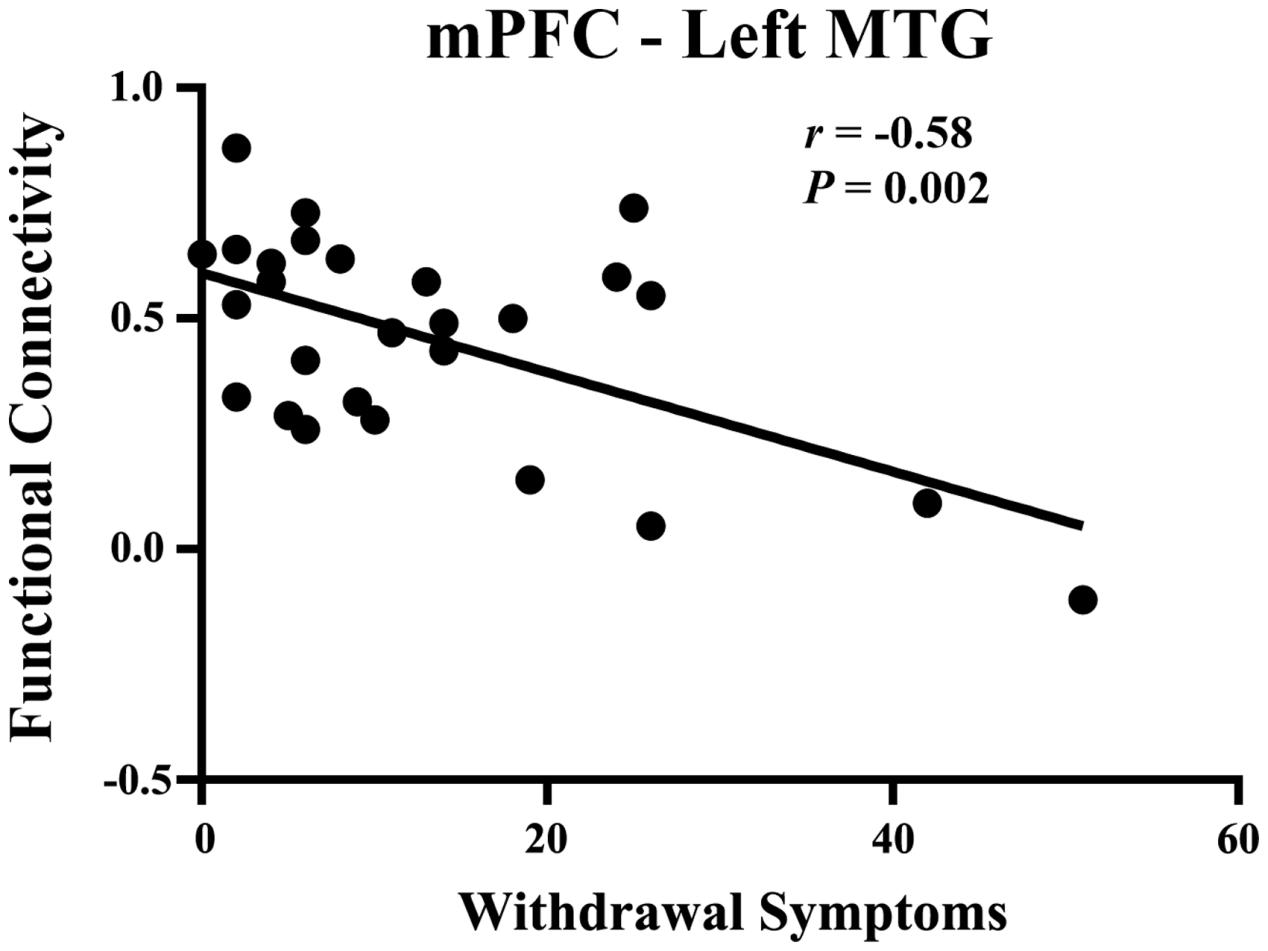
Correlation between withdrawal symptoms and functional connectivity of the mPFC-left MTG in the individuals with HUD after one-year follow-up.

We analyzed the correlations between functional connectivity (between the mPFC and the left MTG, left AI, and right MFG) and psychological characteristics (withdrawal symptoms, anxiety symptoms, depression symptoms, and cravings) at the Baseline and 1 year later. Only the functional connectivity of the mPFC-left MTG was negatively correlated with withdrawal symptoms 1 year later (*r* = −0.58. *p* = 0.002; Bonferroni corrected *p* = 0.05/12 = 0.004; Figure 6).

We did not find a significant correlation between the change in functional connectivity (between the mPFC and the left MTG, left AI, and right MFG) and the total methadone dose during the year (mPFC-left MTG: *r* = −0.138, *p* = 0.517; mPFC-left AI: *r* = −0.037, *p* = 0.827; mPFC-right MFG: *r* = −0.138, *p* = 0.824).

## Discussion

To the best of our knowledge, this is the first longitudinal self-controlled resting-state fMRI study of the effects of MMT on large-scale brain networks in individuals with HUD. The data showed that (1) MMT continuously reduced withdrawal symptoms in individuals with HUD over 1 year of follow-up and (2) the number of relapses were negatively related to the total methadone dose during the one-year period. We also found that (1) MMT enhanced functional connectivity between the mPFC and the left MTG, AI, and right MFG and (2) the mPFC-left MTG connectivity was negatively correlated with the withdrawal symptoms. In this study, the fMRI connectivity data of HC group were just used as referential control and not followed up. Our findings imply that MMT enhanced the internal functional connectivity within the DMN, which was associated with a reduction in withdrawal symptoms in individuals with HUD, and that it enhanced the coupling between the DMN and SN, which might be related to the risk of relapse.

### Methadone dose is negatively associated with numbers of relapses

Our findings are consistent with the results of previous studies (6, 24) showing that higher dosages in MMT resulted in lower levels of relapse. Adequate methadone dosage is a key contributor to optimal treatment outcomes for HUD and is required to alleviate withdrawal symptoms, avoid opioid overdose, and even eliminate persistent illegal opioid use (24). One longitudinal study showed that measures of adequate methadone dose improved addiction severity parameters in opioid substitution treatment (25). These management strategies could optimize treatment outcomes, e.g., by relieving withdrawal symptoms and decreasing cravings for heroin.

### DMN connectivity increased for HUDs on MMT

The DMN (mainly including the mPFC, PCC, and MTG) has been linked to regulation of self-focused thoughts, particularly monitoring psychological states and internal attention, and mediating a dynamic interplay between cognitive functions and emotional processing (26, 27). In addicted individuals, aberrant activation levels within this network have been correlated with a decreased ability, such as deficient tagging of self-relevance and impaired self-awareness (28). A meta-analysis of studies on internet gaming disorder (IGD) showed that individuals with IGD exhibited significant hypoconnectivity within the DMN (29). Low mPFC-MTG connectivity may bias decision-making in smokers, leading to risky behavior and further drug use (30). Internal connectivity within the DMN can influence recall for the best action, including emotional responses to specific events and circumstances from past experiences (31). The mPFC has been shown to be involved in constructing personal meaning from salient stimuli, such as self-reflection, emotion, and evaluation (32). We found that the connectivity between the mPFC and MTG was enhanced and negatively correlated with withdrawal symptoms in individuals with HUD during self-controlled one-year MMT, indicating that the internal connectivity of the DMN was more intense together with improvements in withdrawal symptoms in HUD patients during MMT. Our findings obtained with longitudinal self-controlled MMT provided evidence that MMT may improve monitoring of own psychological states and might benefit emotional regulation by modulating the interconnections of the DMN. The characteristics of connectivity within the DMN might be a feasible neuroimaging biomarker for predicting therapeutic outcomes in individuals with HUD during MMT (33).

### Connectivity between the DMN and SN increased for HUDs on MMT

Different from our hypothesis, our data showed enhanced functional connectivity between the DMN (mPFC) and SN (AI and MFG), consistent with the results of a previous study in which relapsed heroin-dependent individuals showed greater connectivity between the SN and DMN during MMT (16). Similar results were also obtained in another study in which individuals with IGD exhibited significantly enhanced functional connectivity between the DMN and AI (29). The SN participates in processing salient external stimuli, negative coupling with the DMN, and modulating between networks to trigger executive control when a salient stimulation is monitored (29). The relevance of the increased connectivity between the DMN and SN to cognitive impulsivity has been reported previously (34). Given the role of the SN in switching between the DMN and ECN, the increased interaction between DMN and SN might be related to curbing of executive control driven to heightened sensitivity to addiction-related cues and weakened goal-directed behaviors (i.e., inhibitory control). These findings were supported by the results of a systematic review in which MMT was associated with impairments in cognitive functions such as attention, memory, decision-making and emotional interpretation (35). Taken together, the increased connectivity between the DMN and SN might underlie attentional bias to incentive triggers leading to enhanced salience values of heroin cues in individuals with HUD during long-term MMT which in turn could raise relapse risk.

Our previous study showed that (17) for individuals with HUD who did not receive any treatment, methadone can increase functional connectivity between SN and ECNs. These results suggested that methadone might be beneficial to the recovery of the inhibitory control function for individuals with HUD. The present longitudinal self-control study showed that as time went on, long-term MMT program both enhanced the internal functional connectivity within the DMN and the connectivity between the SN and DMN. These results suggest that methadone may have both positive and negative effects on individuals with HUD.

This study had several limitations. First, the urine tests for HUD patients were performed once a month, rather than random multiple tests within a month. This limited our ability to accurately ascertain the number of relapses among patients undergoing MMT. Second, our sample mostly included men because of the difficulties in recruiting female individuals with HUD. Third, although we asked the participants to avoid thinking about anything specific during the resting-state fMRI scans, we could not ensure their compliance with this instruction. However, this may be a communal problem in resting-state fMRI studies. Fourth, we only studied the connectivity of the three core large networks. Other networks may also contribute to the effect of MMT on brain connectivity. Thus, future studies should aim to investigate the characteristics of changes in other large-scale networks. Fifth, we did not collect longitudinal follow-up data of healthy controls. It cannot be excluded that other factors may have an impact on the study results, such as aging of brain function with time, reduced heroin use, less anxiety during scanning, lifestyle changes, health changes, etc.

In conclusion, this longitudinal self-controlled study showed that MMT increased the internal connectivity of the DMN, which was associated with a reduction in withdrawal symptoms in individuals with HUD. MMT also increased the connectivity between the DMN and SN, which might be related to increase in salience values of heroin cues in individuals with HUD during long-term MMT. Our findings suggest that long-term MMT might be a double-edged sword in the treatment of HUD. Thus, more research is needed to identify a balance in guiding MMT protocols to achieve optimal internal and external DMN connectivity.

## Primary finding

1. Long-term methadone maintenance treatment enhanced the connectivity within the default mode network and that between the default mode network and salience network.
2. The connectivity within the default mode network was negatively correlated with the withdrawal symptom score.
3. The number of relapses was negatively correlated to the total methadone dose used during in one-year follow-up.

## Data availability statement

The original contributions presented in the study are included in the article/supplementary material, further inquiries can be directed to the corresponding author/s.

## Ethics statement

The studies involving human participants were reviewed and approved by Tangdu Hospital Ethics Committee. The patients/participants provided their written informed consent to participate in this study.

## Author contributions

JC analyzed the data and drafted the paper. WW, CJ, and QL designed the study and wrote the protocol. YoL, SW, WLi, YaL, LJ, ZL, JZ, FW, WLiu, JX, and HS collected the MRI data. All authors contributed to the article and approved the submitted version.

## Funding

This work was partly funded by Top Talent Foundation of Tangdu Hospital (2019), Discipline innovation and development program of the Second Affiliated Hospital of Air Force Medical University (2021QYJC-003), National Social Science Fund Project (No. 19XXW008), Youth Independent Innovation Science Fund of Tangdu Hospital (2023BTDQN021) and Science and Technology Development Fund of Air Force Military Medical University (2022XC053).

## Conflict of interest

The authors declare that the research was conducted in the absence of any commercial or financial relationships that could be construed as a potential conflict of interest.

## Publisher’s note

All claims expressed in this article are solely those of the authors and do not necessarily represent those of their affiliated organizations, or those of the publisher, the editors and the reviewers. Any product that may be evaluated in this article, or claim that may be made by its manufacturer, is not guaranteed or endorsed by the publisher.

## Footnotes

^1^ http://rfmri.org/dpabi

^2^ https://www.fil.ion.ucl.ac.uk/spm/software/spm12/

^3^ www.mathworks.com

^4^ http://icatb.sourceforge.net
